# A Scoping Review of Strategies and Outcomes of Sleep Education Interventions for Older Adults

**DOI:** 10.1093/geroni/igaf122.3091

**Published:** 2025-12-31

**Authors:** Soeun Jang, Kathy Siepker, Christine Spadola

**Affiliations:** University of Texas at Arlington, The University of Texas at Arlington, Texas, United States; The University of Texas at Arlington, Arlington, Texas, United States; The University of Texas at Arlington, Arlington, Texas, United States

## Abstract

As sleep disturbances are prevalent among older adults, it is well-established that sleep health issues are negatively impacting their physical, cognitive, and emotional well-being. Although sleep health interventions have been developed and tested as a key non-pharmacological strategy to improve sleep quality in older adults, the scope of their educational components and their effectiveness across different delivery modalities remain unclear. We conducted a scoping review to assess the impact of sleep education interventions on older adults’ health and well-being. A search of databases was conducted in September 2024, yielding 1,452 studies published in the past 30 years. After conducting title/abstract and full-text review, 33 studies met our inclusion criteria. The inclusion criteria for included studies were: (1) targeting adults age 60+; (2) including sleep education interventions; and (3) in English; Studies including non-educational or self-guided interventions were excluded. Interventions spanned various countries, with the U.S. (n = 14), followed by Iran, Canada, Japan, and Brazil. Randomized Controlled Trials (RCTs) were the most common (n = 21), followed by quasi-experimental, and pilot studies. Sleep education modalities mostly utilized Cognitive Behavioral Therapy for Insomnia (CBT-I), sleep hygiene education, and relaxation training. CBT-I, interventions that also included physical activity, music, and light therapy showed the greatest improvements in sleep quality and reductions in insomnia severity. Additional benefits from sleep education delivered virtually were also highlighted. Our research highlights that sleep education can be a feasible and effective modality to improve sleep in older adults. Future research should explore tailored interventions for marginalized subgroups of aging populations.

